# Synthesis and Tuberculostatic Activity Evaluation of Novel Benzazoles with Alkyl, Cycloalkyl or Pyridine Moiety

**DOI:** 10.3390/molecules23040985

**Published:** 2018-04-23

**Authors:** Malwina Krause, Henryk Foks, Ewa Augustynowicz-Kopeć, Agnieszka Napiórkowska, Małgorzata Szczesio, Katarzyna Gobis

**Affiliations:** 1 Department of Organic Chemistry, Medical University of Gdańsk, 107 Gen. Hallera Ave., 80-416 Gdańsk, Poland; malwinakrause@gumed.edu.pl (M.K.); hfoks@gumed.edu.pl (H.F.); 2 Department of Microbiology, Institute of Tuberculosis and Pulmonary Diseases, 26 Płocka Str., 01-138 Warsaw, Poland; e.kopec@igichp.edu.pl (E.A.-K.); araceli@op.pl (A.N.); 3 Institute of General and Ecological Chemistry, Technical University of Łodź, Żeromskiego 116 Str., 90-924 Łódź, Poland; malgorzata.szczesio@p.lodz.pl

**Keywords:** benzazole, pyridine, synthesis, tuberculostatic activity, SAR-study, quantum chemical calculations

## Abstract

Compounds possessing benzimidazole system exhibit significant antituberculous activity. In order to examine how structure modifications affect tuberculostatic activity, a series of benzazole derivatives were synthesized and screened for their antitubercular activity. The compounds **1**–**20** were obtained by the reaction between *o*-diamine, *o*-aminophenol, or *o*-aminothiophenol with carboxylic acids or thioamides. The newly synthesized compounds were characterized by IR, ^1^H-NMR, ^13^C-NMR spectra, and elemental analysis. Synthesized benzazoles were evaluated for their tuberculostatic activity toward *Mycobacterium tuberculosis* strains. Quantum chemical calculations were performed to study the molecular geometry and the electronic structure of benzimidazoles GK-151B, **4**, **6**, and benzoxazole **11**, using the Gaussian 03W software (Gaussian, Inc., Wallingford, CT, USA). Three-dimensional structure of benzimidazoles **1**–**3**, MC-9, and GK-151B was determined by ab initio calculation using Gamess-US software. The activity of the received benzimidazoles was moderate or good. All of the benzoxazoles and benzothiazoles demonstrated much lower activity. Benzoxazoles were less active by about 50 times, and benzothiazole by 100 times than the benzimidazole analogs. Quantum chemical calculations showed differences in the distribution of electrostatic potential in the benzazole system of benzimidazoles and benzoxazoles. Three-dimensional structure calculations revealed how the parity of the alkyl substituent at the C2 position impacts the activity. Benzimidazole system is essential for the antituberculosis activity that is associated with the presence of the imine nitrogen atom in N-1 position. Its replacement by an oxygen or sulfur atom results in a decrease of the activity. The parity of the alkyl substituent at the C-2 position also modifies the activity.

## 1. Introduction

Tuberculosis is a disease caused by the presence of *Mycobacterium tuberculosis complex* in humans or animals. This group includes *M. tuberculosis*, *Mycobacterium bovis*, *Mycobacterium bovis BCG*, *Mycobacterium africanum*, and *Mycobacterium microti*. Tuberculosis is transmitted via droplets from one person to another. The disease most commonly affects the lungs, but it can also involve pleura, lymph nodes, bones and joints, central nervous system, and gastrointestinal tract [1]. Tuberculous meningitis is highly dangerous for children and people with untreated human immunodeficiency virus infection. It causes death or disability of about half of the patients [2,3]. Bacteria *M. tuberculosis* initially are present in the lungs, they later penetrate into the brain causing damage [4]. Infections with bacteria of the *Mycobacterium* genus are a concern not only in developing countries, but are a global problem [5]. This results in a continuous search for new drugs and diagnostics, as well as the formation of health programs.

**Figure 1:**
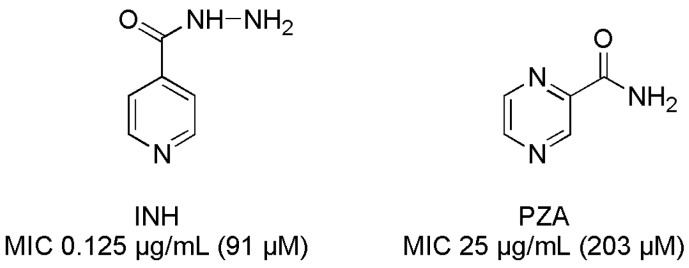
Structure and tuberculostatic activity of isoniazid (INH) and pyrazinamide (PZA).

Many studies support the effect of a heterocyclic moiety with the nitrogen atom on tuberculostatic activity. Such systems include chemotherapeutic agents used clinically, such as isoniazid (INH) and pyrazinamide (PZA) (Figure 1) [6,7]. This fact led the researchers to synthesize a number of pyrazine and pyridine derivatives [8,9]. Compounds possessing other nitrogen heterocyclic systems, like the benzimidazoles, were also obtained [10,11,12,13].

In particular, derivatives that were substituted at the C-2 position with cyclohexylethyl or cyclohexylpropyl moiety were pretty highly active. The compounds were tested against three strains of *M. tuberculosis*: a standard strain called H_37_Rv and two strains isolated from patients, namely Spec. 210-resistant to *p*-aminosalicylic acid, isoniazid, rifampicin, and ethambutol and Spec. 192-sensitive to all medications. Tuberculostatic activity of these compounds was expressed as minimum inhibitory concentration (MIC) value in the range of 6.2–25 µg/mL [14].

The high activity of compounds with cyclohexylethyl substituent at the benzimidazole structure encouraged us to synthesize other heterocyclic systems possessing that moiety. Among the compounds that were obtained were derivatives of 1,3,4-oxadiazole-2-thione, 1,2,4-triazole-5-thione, and benzimidazole-like systems. However, tuberculostatic activity was shown only by benzimidazole analogs. The most active were naphtho[*d*]imidazole, imidazole[*d*]pyridine, and imidazole[*d*]phenazine. MIC values for those compounds ranged from 1.5 to 3.1 µg/mL. All of the obtained compounds exhibited higher activity than the pyrazinamide, a first line antituberculosis drug [15].

**Figure 2:**
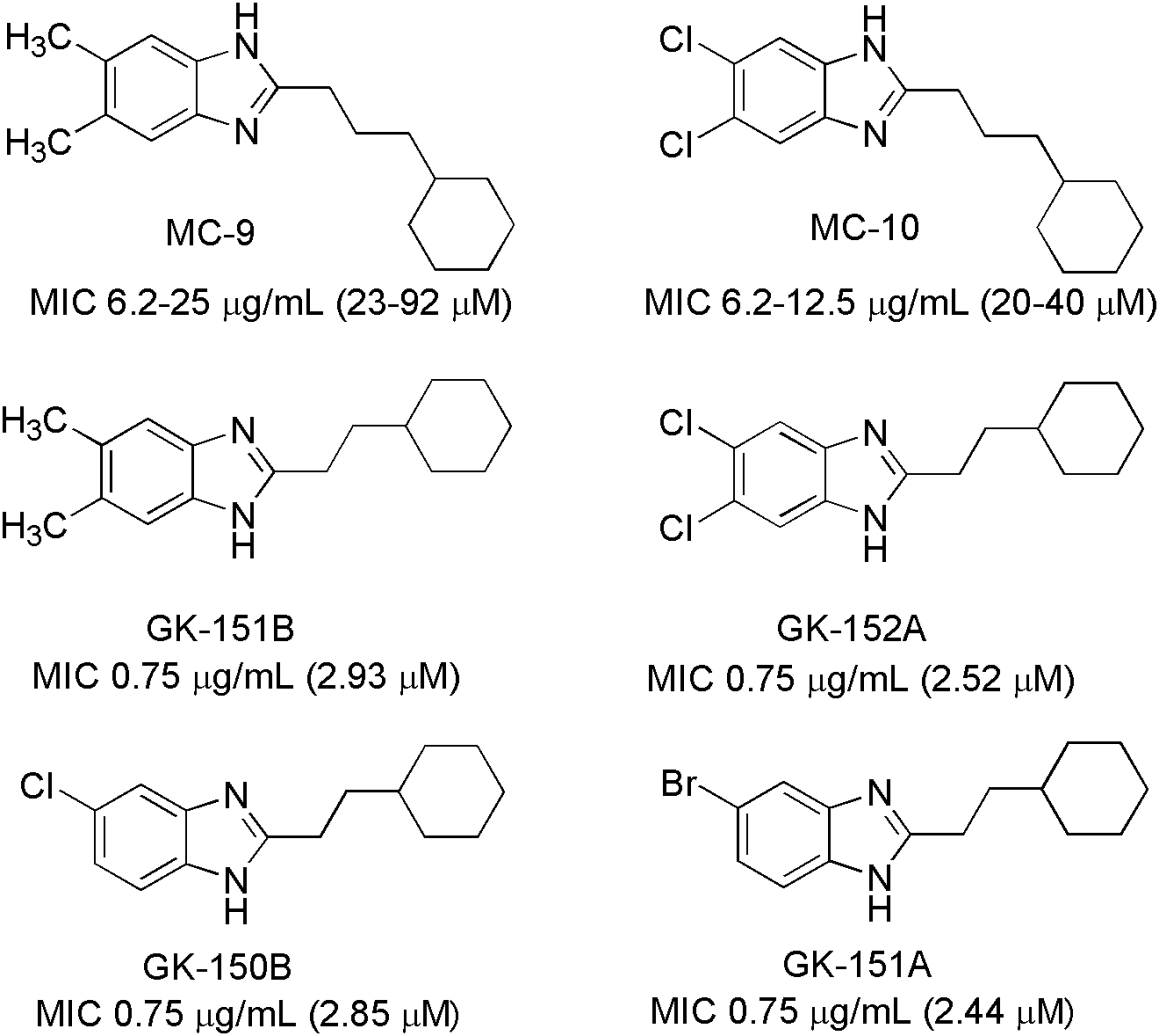
Structure and tuberculostatic activity of some benzimidazole derivatives.

Subsequent studies gave derivatives of 2-(2-cyclohexylalkyl)-1*H*-benzo[*d*]imidazole. Among them, there were the most active compounds that contained two methyl groups in C-5 and C-6 positions of the benzimidazole system (Figure 2). The MIC values of these compounds were 0.8–6.2 µg/mL [16,17]. At the same time, the compounds exhibited low cytotoxicity against human epidermal neonatal fibroblasts (ATCC PCS-201-010) [15,17]. Thus, because of their high tuberculostatic activity and low cytotoxicity these compounds were considered as very good candidates for tuberculosis drugs, although the mechanism of their action remained unknown.

It inspired further studies on the effect of replacing one of the nitrogen atoms of the benzimidazole by an oxygen or sulfur atom on tuberculostatic activity. In particular, the antituberculosis activity of some benzoxazoles [18,19,20,21,22] and benzothiazoles [23,24,25,26] has been described previously by other authors.

The purpose of this study was to obtain derivatives of 1*H*-benzo[*d*]imidazole, benzo[*d*]oxazole, and benzo[*d*]thiazole that were substituted at the C-2 and C-5 positions. The receipt of these compounds used cyclization reactions of carboxylic acids, 4-substituted pyridine-2-carbothioamide or picolinonitrile with *o*-phenylenediamines, *o*-aminophenols, or *o*-aminothiophenols. Substituent in the C-2 position differed in the length of the alkyl chain, in the presence of cyclic ring and in their saturation.

C-5 position of the benzazole system occurred in bromine, chlorine or fluorine atom, and in the methyl group or trifluoromethyl group. Substrates have been selected in such a way that the resulting products were derivatives or structural analogs of benzimidazoles, for which the highest results of tuberculostatic activity have been determined [16]. Carboxylic acids with different chain lengths were used to determine its effect on potency. We wanted to determine, how the exchange of the NH group in position 1 of the benzimidazole system by an oxygen or sulfur atom would affect tuberculostatic activity. In addition, we wanted to verify the effect of the presence of various cyclic substituents, such as cyclohexyl, pyridyl, and phenyl rings on the antitubercular activity, and to identify a cyclic ring that promotes the occurrence of tuberculostatic activity.

## 2. Results and Discussion

### 2.1. Chemistry

**Scheme 1:**
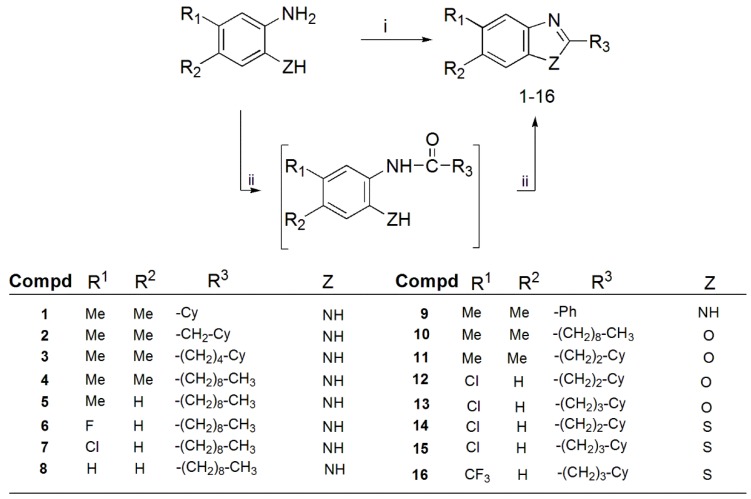
Synthesis of benzimidazoles **1**–**9**, benzoxazoles **10**–**13,** and benzothiazoles **14**–**16.**

Initially, benzimidazoles **1**–**8** were obtained using the method that was previously developed by our team [16]. The starting materials were fused at 160–180 °C without the presence of a solvent (Scheme 1). The applied method provided products in good or very good yields of up to 46–80%. Whereas, benzimidazole **9** was obtained by heating it in the presence of polyphoshoric acid at 160 °C for 12 h.

Subsequently, the synthesis of benzoxazoles **10**–**11** was performed. The starting materials reacted in the environment of polyphosphoric acid at a temperature of 160 °C. Reactions were performed for 4 h. The yield of the reaction varied widely, ranging from 47% to 77%.

Then, the benzoxazole derivatives **12**–**13** were obtained. 2-Amino-4-chlorophenol was used in their synthesis by the method of Kaul, which had previously been used for the synthesis of benzimidazoles. Contrary to the claims by the authors about the cyclization of reagents to the benzimidazole or benzoxazole system, only acid amides were obtained [27]. The amides were then heated with polyphosphoric acid (PPA) analogously for cyclization as compounds **10**–**11**. The compounds were obtained with a yield of 25–71%.

For the synthesis of compounds **14**–**16**, a method that was developed by researchers at the University of Shiraz was used [28]. 2-Amino-4-chlorothiophenol or 2-amino-4-trifluoromethylthiophenol and the appropriate carboxylic acid with methanesulfonic acid (MSA) and the silica gel were heated at 140 °C for 72 h. Under these conditions, the yields of intended compounds were 17–65%.

**Scheme 2:**
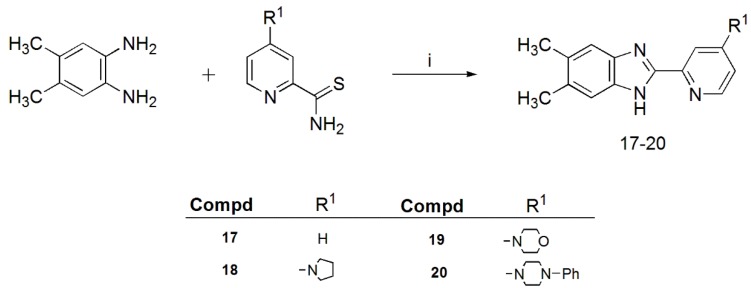
Synthesis of 2-pyridin-2-yl-1H-benzoimidazole derivatives **17**–**20.**

Thioamide derivatives were used to obtain benzimidazoles possessing pyridine moiety **17**–**20** (Scheme 2). When 4,5-dimethylbenzene-1,2-diamine was heated with the appropriate thioamide in the presence of ethylene glycol for 2 h, pyridine derivatives were obtained with a good yield in the range 70–94%.

All if the newly synthesized compounds as well as already known [29,30,31] were characterized by elemental analysis, IR, ^1^H-NMR, and ^13^C-NMR spectra. 

### 2.2. Tuberculostatic Activity

The obtained benzazole derivatives **1**–**20** were evaluated for their in vitro tuberculostatic activity against the *M. tuberculosis* H_37_Rv strain and a “wild” strain isolated from tuberculosis patients (Spec. 210) resistant to *p*-aminosalicylic acid (PAS), INH, ethambutol (ETB), and rifampicin (RMP). 

The MIC values were determined as the minimum concentration inhibiting the growth of tested tuberculosis strains in relation to the probe with no tested compound. INH, PZA, and RMP were used as reference drugs. The results were summarized in Table 1.

The resulting benzimidazoles **1**–**9** were characterized by very good to weak tuberculostatic activity (3.1–100 µg/mL equivalent to 10–409 µM). The studies revealed that benzimidazole derivatives without linker (**1**) or with even aliphatic chain (**3**, **4**) at the C-2 position exhibited the highest antitubercular activity with MIC values that were ranging from 1.5 to 3.1 µg/mL (5–13 µM). If the linker contains an odd number of carbon atoms (**2**) the activity is more than two fold lower (MIC 6.25–25 µM). Replacement of the cyclohexyl ring to the aliphatic chain does not cause significant changes in the activity. 

As we have observed previously, compounds that contained two methyl groups at the C-5 and C-6 positions demonstrated higher potency. Benzimidazole **6** with high electronegative fluorine atom showed lower activity (MIC 12.5–25 µg/mL equivalent 95–190 µM). The absence of substituents on the phenyl ring (**8**) results in a complete loss of activity. The results of the biological tests that were obtained for the group of benzimidazole analogs were much lower than the results of corresponding benzimidazoles [16,17]. Benzoxazoles **10**–**11** possessing methyl group at C-5 and C-6 position showed lower activity. These compounds were active at the level corresponding to MIC value of 25–50 µg/mL (91–194 µM). Benzoxazole **11**, which is an analog of the previously described benzimidazole **GK-151B** that exhibits a significant selectivity against *M. tuberculosis* [16], showed much lower activity. A weaker activity was also exhibited by benzoxazoles **12**–**13** substituted by a chlorine atom in the same position with MICs 50–100 µg/mL (190–389 µM). Benzothiazoles **14**–**16** also showed the same level of activity. 

Although the antitubercular activity of benzimidazoles **17**–**20** possessing pyridine ring is moderate (MIC 12.5–25 µg/mL), they exhibit lower MIC value than the derivatives with alkyl chain or cyclohexyl ring. By comparing the tuberculostatic activity of compounds that differ in the type of the ring at position C2, a significant increase in the activity was observed for derivative **1** with cyclohexyl ring. Compounds possessing aryl group (**9**, **17**) exhibited weaker activity (MICs 12.5–50 µg/mL). However, derivative **17** with pyridine ring showed four times higher antitubercular activity than the phenyl derivative (**9**).

### 2.3. Quantum Chemical Calculations

**Figure 3:**
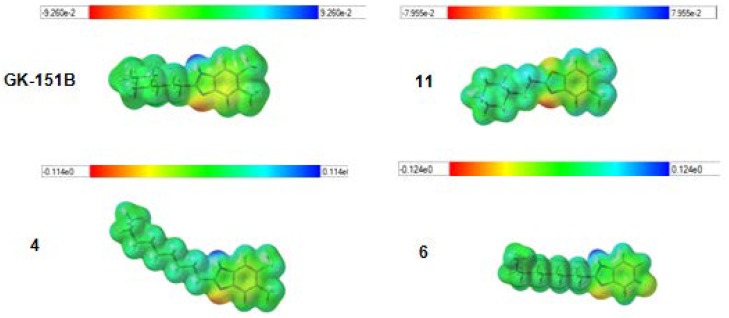
Molecular electrostatic potential (MEP) maps on the isodensity surface calculated at the B3LYP/6-31G(d).

Density functional theory calculations for structures of benzimidazole **GK-151B**, **4**, **6,** and benzoxazole **11** were performed (Figure 3). Electrostatic potential surfaces were plotted while employing 6-31G(d) basis set, using the computer software GaussView and Gaussian 03W. On the contained maps the red region indicates the region of the greatest electron density, while the structural fragment with the lowest electron density corresponds to the blue region. All of the tested benzimidazole derivatives have a similarly distributed positive charge. The positive charge appears to be localized mostly at the hydrogen atom that was connected with nitrogen atom in the N-1 position. In the case of benzimidazole **6**, strongly electronegative fluorine atom causes the mild effect on the charge distribution in the ring. Benzoxazole **11** exhibits a significantly different charge distribution when compared to benzimidazole derivatives. The high positive region does not occur. 

Just the electronegative region that is caused by the presence of oxygen and nitrogen atoms was observed. Table 2 shows the calculated value of dipole moment (μ) and the partition coefficient (log P). The lower tuberculostatic activity of benzimidazole **4** as compared to the compound **GK-151B** may be related to their higher value of LogP (5.95). The high value of LogP causes stronger interaction between the cell membrane and impediments in reaching the molecular target [32,33]. The high dipole moment (4.92 D) of compound **6** could likely be a reason of poor penetration into cells [34]. While comparing the last two magnitudes for compounds **GK-151B** and **11**, we can conclude that benzoxazole with a smaller dipole moment (0.93 D) and with the similar value of log P (5.02), assuming transport by free diffusion, should penetrate into the cells faster than the benzimidazole. Thus, much lower activity of compound **11** is probably not due to its weak penetration into bacilli, but due to its poor interaction with the molecular target. Analysis of calculated quantities and the charge distributions allow for implying that the NH fragment is involved in the target binding and is essential for tuberculostatic activity.

**Figure 4:**
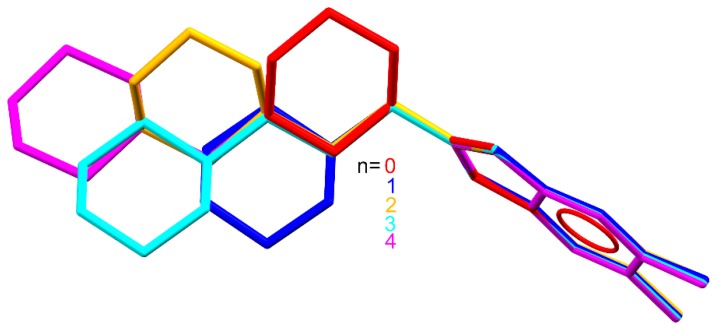
Spatial arrangement of benzimidazoles with a length of linker *n* = 0–4.

The three-dimensional structure of benzimidazoles **1**–**3**, **GK-151B**, and **MC-9** was determined to check how the parity of aliphatic chain at C2 position affect the antitubercular activity. Quantum chemical calculations were performed using the DFT/M06-2X/6-311G(d,p) method that is available in the software Gamess-US. Calculations were conducted for five compounds with a length of linker *n* = 0–4. A similar arrangement of molecules in the space for compounds possessing even linker has been observed (**1**, **3**, **GK-151B**), while compounds having an odd chain are arranged in a different way (**2**, **MC-9**) (Figure 4). Comparison of the obtained results with antimycobacterial activity confirmed our theory that the lack of a linker or the presence of an even aliphatic chain at the C2 position is important for increasing the antitubercular activity. Performed studies suggest that the substituent at the C2 position of the benzimidazole ring participates in the process of adjusting to the cell target.

## 3. Materials and Methods 

### 3.1. Chemistry

All of the materials and solvents were of analytical reagent grade. Thin-layer chromatography was performed on Merck (Darmstadt, Germany) silica gel 60F_254_ plates and visualized with UV. The stationary phase for column chromatography was silica gel (60 Å, 70–230 mesh). The results of elemental analyses (%C, H, N) for all of the obtained compounds that were determined on Perkin-Elmer PE 2400 Series II CHNS analyzer (Perkin-Elmer, Shelton, CT, USA) were in agreement with the calculated values within ±0.4% range. ^1^H- and ^13^C-NMR spectra in CDCl_3_ or DMSO-*d*_6_ were recorded on Varian Unity Plus (500 MHz) and Varian Gemini (200 MHz) instruments (Varian, Palo Alto, CA, USA). ^1^H-NMR spectra of selected compounds can be found in Supplementary Materials. IR Spectra (KBr) were determined as KBr pellets of the solids on a Satellite FT-IR spectrophotometer (Mattson Instruments, Madison, WI, USA). Melting points were determined on a Stuart SMP30 apparatus and were uncorrected (Bibby Scientific, Staffordshire, UK). 

#### 3.1.1. General Procedure for the Synthesis of Benzimidazoles **1**–**8**

Appropriate carboxylic acid (5.5 mmol) and appropriate diamine (5 mmol) were heated on Wood’s metal bath at 160–180 °C for 1 h. While cooling down. A 10% NaOH solution (10 mL) was added and the reaction mixture was stirred at room temperature for 24 h. Then, the precipitated benzimidazoles were filtered off, washed with water to the neutral reaction, dried, and purified by recrystallization from the methanol—water mixture 2:1 with the addition of activated carbon.

*2-Cyclohexyl-5,6-dimethyl-1H-benzo[d]imidazole* (**1**). Starting from cyclohexanecarboxylic acid (0.704 g) and 4,5-dimethylbenzene-1,2-diamine (0.680 g), the title compound **1** was obtained as beige crystals (0.812 g, 71%): m.p. 226–228 °C; IR (KBr): 2924, 2855 (ν C–H), 1539 (δ N–H), 1449 (ν C=C), 1307 (ν C–C), 848 (γ C–H) cm^−1^; ^1^H-NMR (500 MHz, DMSO-*d*_6_): δ 1.21–1.78 (m, 8H, 4CH_2_), 1.95–1.98 (m, 2H, CH_2_), 2.25 (s, 6H, 2CH_3_), 2.74–2.78 (m, 1H, CH), 7.19 (s, 2H, ArH), 11.50 (br s, 1H, NH) ppm; Anal. Calcd. for C_15_H_20_N_2_ (228.16): C, 78.90; H, 8.83; N, 12.27; Found: C, 79.17; H, 8.90; N, 12.34 [29].

*2-(Cyclohexylmethyl)-5,6-dimethyl-1H-benzo[d]imidazole* (**2**). Starting from 2-cyclohexylacetic acid (0.782 g) and 4,5-dimethylbenzene-1,2-diamine (0.680 g), the title compound **2** was obtained as beige crystals (0.784, 65%): m.p. 196–198 °C; IR (KBr): 2292, 2849 (ν C–H), 1543 (δ N–H), 1448, 1410 (ν C=C), 1300 (ν C–C), 1003 (δ C–H), 843 (γ C–H) cm^−1^; ^1^H-NMR (500 MHz, DMSO-*d*_6_): δ 0.91–1.21 (m, 5H, 2CH_2_ and CH), 1.57–1.79 (m, 6H, 3CH_2_), 2.25 (s, 6H, 2CH_3_), 2.55 (d, 2H, CH_2_, *J* = 7 Hz), 7.18 (s, 2H, ArH), 11.50 (br s, 1H, NH) ppm; Anal. Calcd. for C_16_H_22_N_2_ (242.18): C, 79.29; H, 9.15; N, 11.56; Found: C, 79.03; H, 8.99; and, N, 11.41.

*2-(4-Cyclohexylbutyl)-5,6-dimethyl-1H-benzo[d]imidazole* (**3**). Starting from 5-cyclohexylpentanoic acid (1.056 mL) and 4,5-dimethylbenzene-1,2-diamine (0.680 g), the title compound **3** was obtained as beige crystals (1.104 g, 78%): m.p. 112–113 °C; IR (KBr): 2922, 2850 (ν C–H), 1539 (δ N–H), 1463, 1447 (ν C=C), 1308 (ν C–C), 1020, 999 (δ C–H), 852 (γ C–H) cm^−1^; ^1^H-NMR (500 MHz, DMSO-*d*_6_): δ 0.76–0.83 (m, 2H, CH_2_), 1.05–1.29 (m, 8H, 4CH_2_), 1.56–1.71 (m, 7H, 3CH_2_ and CH), 2.25 (s, 6H, CH_3_), 2.71 (t, 2H, CH_2_, *J* = 7 Hz), 7.18 (s, 2H, ArH), 11.90 (br s, 1H, NH) ppm; Anal. Calcd. for C_19_H_28_N_2_ (284.23): C, 80.23; H, 9.92; N, 9.85; Found: C, 79.97; H, 10.02; and, N, 9.71.

*5,6-Dimethyl-2-nonyl-1H-benzo[d]imidazole* (**4**). Starting from decanoic acid (0.946 g) and 4,5-dimethyl-benzene-1,2-diamine (0.680 g), the title compound **4** was obtained as beige crystals (1.091 g, 80%): m.p. 71–73 °C; IR (KBr): 2927, 2851 (ν C–H), 1542 (δ N–H), 1466, 1411 (ν C=C), 1307 (ν C–C), 998 (δ C–H), 854, 724 (γ C–H) cm^−1^; ^1^H-NMR (200 MHz, DMSO-*d*_6_): δ 0.80–0.86 (m, 3H, CH_3_), 1.22 (s, 12H, 6CH_2_), 1.68–1.74 (m, 2H, CH_2_), 2.26 (s, 6H, 2CH_3_), 2.73 (t, 2H, CH_2_, *J* = 7 Hz), 7.15 (s, 1H, ArH), 7.25 (s, 1H, ArH), 11.89 (s, 1H, NH) ppm; Anal. Calcd. for C_18_H_28_N_2_ (272.23): C, 79.36; H, 10.36; N, 10.28; Found: C, 79.47; H, 10.45; and, N, 10.16.

*5-Methyl-2-nonyl-1H-benzo[d]imidazole* (**5**). Starting from decanoic acid (0.946 g) and 4-methylbenzene-1,2-diamine (0.610 g), the title compound **5** was obtained as brown crystals (0.801 g, 62%): m.p. 74–75 °C; IR (KBr): 2921, 2852 (ν C–H), 1547 (δ N–H), 1448, 1421 (ν C=C), 1281 (ν C–C), 803 (γ C–H) cm^−1^; ^1^H-NMR (200 MHz, DMSO-*d*_6_): δ 0.81–0.86 (m, 3H, CH_3_), 1.22 (s, 12H, 6CH_2_), 1.73–1.76 (m, 2H, CH_2_), 2.37 (s, 3H, CH_3_), 2.75 (t, 2H, CH_2_, *J* = 7 Hz), 6.88 (d, 1H, ArH, *J* = 8 Hz), 7.22–7.33 (m, 2H, ArH), 12.00 (s, 1H, NH) ppm; Anal. Calcd. for C_17_H_26_N_2_ (258.21): C, 79.02; H, 10.14; N, 10.84; Found: C, 79.15; H, 10.40; and, N, 11.00 [30].

*5-Fluoro-2-nonyl-1H-benzo[d]imidazole* (**6**). Starting from decanoic acid (0.946 g) and 4-fluorobenzene-1,2-diamine (0.630 g), the title compound **6** was obtained as light beige crystals (0.732 g, 56%): m.p. 59–60 °C; IR (KBr): 2923, 2852 (ν C–H), 1539 (δ N–H), 1487, 1456, 1421 (ν C=C), 1139, 958 (δ C–H), 843, 801 (γ C–H) cm^−1^; ^1^H-NMR (200 MHz, DMSO-*d*_6_): δ 0.80–0.83 (m, 3H, CH_3_), 1.22–1.26 (m, 12H, 6CH_2_), 1.70–1.73 (m, 2H, CH_2_), 2.77 (t, 2H, CH_2_, *J* = 7 Hz), 6.89–6.98 (m, 1H, ArH), 7.24–7.41 (m, 2H, ArH), 12.27 (s, 1H, NH) ppm; Anal. Calcd. for C_16_H_23_N_2_F (262.18): C, 73.25; H, 8.84; N, 10.68; Found: C, 73.28; H, 9.06; and, N, 10.59.

*5-Chloro-2-nonyl-1H-benzo[d]imidazole* (**7**). Starting from decanoic acid (0.946 g) and 4-chlorobenzene-1,2-diamine (0.713 g), the title compound **7** was obtained as light brown crystals (0.896 g, 64%): m.p. 66–68 °C; IR (KBr): 2953, 2923 (ν C–H), 1540 (δ N–H), 1468, 1447, 1413 (ν C=C), 1292 (ν C–C), 924 (δ C–H), 854, 801 (γ C–H) cm^−1^; ^1^H-NMR (200 MHz, DMSO-*d*_6_): δ 0.80–0.83 (m, 3H, CH_3_), 1.22 (s, 12H, 6CH_2_), 1.70–1.73 (m, 2H, CH_2_), 2.78 (t, 2H, CH_2_, *J* = 7 Hz), 7.10 (d, 1H, ArH, *J* = 8 Hz), 7.39–7.51 (m, 2H, ArH), 12.35 (s, 1H, NH) ppm; Anal. Calcd. for C_16_H_23_N_2_Cl (278.15): C, 68.92; H, 8.31; N, 10.05; Found: C, 69.04; H, 8.36; and, N, 9.85 [30].

*2-Nonyl-1H-benzo[d]imidazole* (**8**). Starting from decanoic acid (0.946 g) and benzene-1,2-diamine (0.540 g), the title compound **8** was obtained as light brown crystals (0.564 g, 46%): m.p. 124–125 °C; IR (KBr): 2924, 2852 (ν C–H), 1541 (δ N–H), 1454, 1421 (ν C=C), 1271 (ν C–C), 752, 739 (γ C–H) cm^−1^; ^1^H-NMR (200 MHz, DMSO-*d*_6_): δ 0.81–0.84 (m, 3H, CH_3_), 1.23–1.27 (m, 12H, 6CH_2_), 1.71–1.78 (m, 2H, CH_2_), 2.78 (t, 2H, CH_2_, *J* = 7 Hz), 7.03–7.14 (m, 2H, ArH), 7.43–7.45 (m, 2H, ArH), 12.16 (s, 1H, NH) ppm; Anal. Calcd. For C_16_H_24_N_2_ (244.19): C, 78.64; H, 9.90; N, 11.46; Found: C, 78.69; H, 10.14; and, N, 11.48 [30].

#### 3.1.2. General Procedure for Synthesis of Benzimidazole **9**

Benzoic acid (1.1 mmol, 0.134 g) and 1,2-diamine-4,5-dimethylbenzen (1 mmol, 0.136 g) and PPA (2 g) were heated in the oil bath at the 160 °C for 12 h. While cooling down, a 10% NaOH solution (5 mL) was added. The precipitated product was filtered off, washed with water to the neutral reaction, dried, and purified by recrystallization from the dioxin—water mixture 1:1 with the addition of activated carbon.

*5,6-dimethyl-2-phenyl-1H-benzo[d]imidazole* (**9**). The title compound was obtained as beige crystals (0.162 g, 73%) m.p. 250–252 °C; IR (KBr): 3057 (ν N–H), 2973, 2925 (ν C–H), 1599 (δ N–H), 1560 (ν C=N), 1493 (ν C=C), 730 (γ C–H) cm^−1^; ^1^H-NMR (500 MHz, DMSO-*d*_6_ + D_2_O): δ 2.31 (s, 6H, CH_3_), 7.37 (s, 2H, ArH), 7.46–7.54 (m, 3H, ArH), 8.13 (d, 2H, ArH, *J* = 7 Hz) ppm; ^13^C-NMR (125 MHz, DMSO-*d*_6_): δ 20.49 (2C), 126.76 (2C), 129.03 (2C), 129.38 (2C), 129.72 (2C), 130.17, 131.35, 133.33, 150.56, 167.79 ppm; Anal. Calcd. for C_15_H_14_N_2_ (222.12): C, 81.05; H, 6.35; N, 12.60; Found: C, 81.36; H, 6.09; and, N, 12.24 [29].

#### 3.1.3. General Procedure for Synthesis of 4,5-Dimethylbenzoxazoles **10**, **11**

Appropriate carboxylic acid (5.5 mmol), 2-amino-4,5-dimethylphenol (5 mmol, 0.690 g) and PPA (10 g) were heated in the oil bath at 160 °C for 4 h. While cooling down, a 10% NaOH solution (10 mL) was added. The mixture was extracted with benzene (3 × 30 mL), dried with MgSO_4_, and evaporated. Crude oily products were purified by column chromatography with methylene chloride (**9**) or chloroform (**10**) as the eluent.

*5,6-Dimethyl-2-nonylbenzo[d]oxazole* (**10**). Starting from decanoic acid (0.946 g), the title compound **10** was obtained as light beige crystals (1.044 g, 76%): m.p. 25–26 °C; IR (KBr): 2925, 2854 (ν C–H), 1574 (δ N–H), 1466 (ν C=C), 1282 (ν C–O–Ar), 1148 (δ C–H), 868, 849 (γ C–H) cm^−1^; ^1^H-NMR (500 MHz, DMSO-*d*_6_): δ 0.82 (t, 3H, CH_3_, *J* = 7 Hz), 1.20–1.24 (m, 12H, 7CH_2_), 1.71–1.74 (m, 2H, CH_2_), 2.26 (s, 3H, CH_3_), 2.28 (s, 3H, CH_3_), 2.84 (t, 2H, CH_2_, *J* = 7 Hz), 7.39 (s, 2H, ArH) ppm; ^13^C-NMR (125 MHz, DMSO-*d*_6_): δ 14.61, 20.31, 20.57, 22.78, 26.85, 28.37, 29.12, 29.31, 29.33, 29.53, 31.94, 111.23, 119.85, 133.02, 133.83, 139.76, 149.50, 166.69 ppm; molecular weight for C_18_H_27_NO (273.21); and, MS (+): *m*/*z* = 274 (100%, [M + H]^+^), *m*/*z* = 275 (20%, [M + 2H]^+^);

*2-(2-Cyclohexylethyl)-5,6-dimethylbenzo[d]oxazole* (**11**). Starting from 3-cyclohexylpropionic acid (0.942 mL), the title compound **11** was obtained as light beige solid (0.869 g, 68%): m.p. 49–51 °C; IR (KBr): 2923, 2850 (ν C–H), 1572 (ν C=N), 1464, 1451 (ν C=C), 1276 (ν C–O–Ar), 1146 (δ C–H), 871 (γ C–H) cm^−1^; ^1^H-NMR (500 MHz, DMSO-*d*_6_): δ 0.87–0.89 (m, 2H, CH_2_), 1.12–1.16 (m, 4H, 2CH_2_), 1.62–1.72 (m, 7H, 3CH_2_ and CH), 2.27 (s, 3H, CH_3_), 2.29 (s, 3H, CH_3_), 2.86 (t, 2H, CH_2_, *J* = 7.8 Hz), 7.40 (s, 2H, ArH) ppm; ^13^C-NMR (125 MHz, DMSO-*d*_6_): δ 20.3, 20.6, 26.0, 26.4 (2C), 26.7, 33.1 (2C), 34.3, 37.2, 111.3, 119.9, 133.1, 133.9, 139.8, 149.5, 167.0 ppm; Anal. Calcd. for C_17_H_23_NO (257.18): C, 79.33; H, 9.01; N, 5.44; Found: C, 79.34; H, 9.29; and, N, 5.12 [31].

#### 3.1.4. General Procedure for the Synthesis of 5-Chlorobenzoxazoles **12**, **13**

Into the pressure equalizing dropping funnel 5 mL of anhydrous toluene, 1 mL of anhydrous DMF (13 mmol) and 0.8 mL of thionyl chloride (11 mmol) were poured. After 5 min, the separated lower layer (*N*,*N*-dimethylchlorosulfitemethaniminium chloride) was added drop wise to a solution of the carboxylic acid (5 mmol) in 10 mL of anhydrous methylene chloride. After a further 15 min, 2-amino-4-chlorophenol (5 mmol, 0.718 g) was scattered to a solution followed by drop wise adding of 6 mL of methylene chloride and 2.5 mL of anhydrous pyridine (15 mmol) mixture. The solution was stirred at room temperature for 18 h. The solution was washed with distilled water (3 × 15 mL) and dried over anhydrous MgSO_4_. The drying agent was filtered off and the solvent evaporated. The resulting amide was cyclized without further purification by adding PPA (10 g) and heating on an oil bath at 160 °C for 4 h. Then, cooled and basified with 10% NaOH. The alkalized solution was extracted with benzene (3 × 30 mL). The combined organic layers were dried over anhydrous MgSO_4_. The drying agent was filtered off and the solvent evaporated. The resulting product was purified by column chromatography eluting with chloroform (**12**) or methylene chloride (**13**).

*5-Chloro-2-(2-cyclohexylethyl)benzo[d]oxazole* (**12**). Starting from 3-cyclohexylpropionic acid (0.857 mL), the title compound **12** was obtained as colourless crystals (0.401 g, 30%): m.p. 43–44 °C; IR (KBr): 3096–3047 (ν C–H), 2925, 2852 (ν C–H), 1567 (ν C=N), 1453, 1426 (ν C=C), 1257 (ν C–O–Ar), 1051 (ν C–Cl), 810 (γ C–H) cm^−1^; ^1^H-NMR (200 MHz, DMSO-*d*_6_): δ 0.87–1.29 (m, 6H, 3CH_2_), 1.63–1.74 (m, 7H, 3CH_2_ and 1H CH), 2.94 (t, 2H, CH_2_, *J* = 7 Hz), 7.36–7.42 (m, 1H, ArH), 7.68–7.79 (m, 2H, ArH) ppm; Anal. Calcd. for C_15_H_18_ClNO (263.11): C, 68.30; H, 6.88; N, 5.31; Found: C, 68.30; and, H, 6.73; N, 5.26 [3].

*5-Chloro-2-(3-cyclohexylpropyl)benzo[d]oxazole* (**13**). Starting from 4-cyclohexylbutyric acid (0.850 g), the title compound **13** was obtained as rufous crystals (0.943 g, 68%): m.p. 33–35 °C; IR (KBr): 2923, 2850 (ν C–H), 1568 (ν C=N), 1451 (ν C=C), 1256 (ν C–O–Ar), 1168 (δ C–H), 1054 (ν C–Cl), 801 (γ C–H) cm^−1^; ^1^H-NMR (200 MHz, CDCl_3_): δ 0.90–1.45 (m, 8H, 4CH_2_), 1.67–1.90 (m, 7H, 3CH_2_ and 1H CH), 2.89 (t, 2H, CH_2_, *J* = 8 Hz), 7.23–7.64 (m, 3H, ArH) ppm; Anal. Calcd. for C_16_H_20_ClNO (277.12): C, 69.18; H, 7.26; N, 5.04; Found: C, 68.98; H, 7.63; and, N, 5,30.

#### 3.1.5. General Procedure for the Synthesis of Benzothiazoles **14**, **15**

2-Amino-4-chlorothiophenol (5 mmol, 0.798 g) and the appropriate carboxylic acid (5 mmol) were heated with silica gel (1.5 g) and methanesulfonic acid (5 mL) at 140 °C for 72 h. After this time, the cooled reaction mixture was diluted with 100 mL of ethyl acetate and silica gel was filtered off by gravity. The solution was washed with 5% NaHCO_3_ (3 × 30 mL) until alkaline. The organic layer was dried over anhydrous MgSO_4_. The drying agent was filtered off and the solvent evaporated. The resulting product was purified by column chromatography eluting with chloroform (**14**) or methylene chloride (**15**).

*5-Chloro-2-(2-cyclohexylethyl)benzo[d]thiazole* (**14**). Starting from 3-cyclohexylpropionic acid (0.857 mL), the title compound **14** was obtained as green crystals (0.240 g, 17%): m.p. 43–45 °C; IR (KBr): 2923, 2851 (ν C–H), 1587 (ν C=N), 1516, 1440 (ν C=C), 1074 (ν C–Cl), 799 (γ C–H) cm^−1^; ^1^H-NMR (200 MHz, DMSO-*d*_6_): δ 1.31–1.76 (m, 13H, 12H 6CH_2_ and 1H CH), 3.11 (t, 2H, CH_2_, *J* = 8 Hz), 7.41–7.47 (m, 1H, ArH), 7.99–8.09 (m, 2H, ArH) ppm; ^13^C-NMR (50 MHz, DMSO-*d*_6_): δ 25.7 (2C), 26.0, 31.0, 32.4 (2C), 36.4, 36.5, 121.5, 123.5, 124.8, 130.7, 133.3, 153.7, 174.7 ppm; Anal. Calcd. for C_15_H_18_ClNS (279.08): C, 64.38; H, 6.48; N, 5.01; Found: C, 64.50; H, 6.49; N, 4.87.

*5-Chloro-2-(3-cyclohexylpropyl)benzo[d]thiazole* (**15**). Starting from 4-cyclohexylbutyric acid (0.850 g), the title compound **15** was obtained as colourless crystals (0.937 g, 64%): m.p. 42–46 °C; IR (KBr): 2922, 2849 (ν C–H), 1517, 1436 (ν C=C), 1070 (ν C–Cl), 797 (γ C–H) cm^−1^; ^1^H-NMR (200 MHz, DMSO-*d*_6_): δ 0.81–1.28 (m, 9H, 8H 4CH_2_ and 1H CH), 1.63–1.87 (m, 8H, 4CH_2_), 7.42–7.48 (m, 1H, ArH), 8.00–8.11 (m, 2H, ArH) ppm; ^13^C-NMR (50 MHz, DMSO-*d*_6_): δ 26.2 (2C), 26.5, 26.7, 33.1 (2C), 34.1, 36.6, 37.1, 122.0, 123.9, 125.2, 130.4, 133.4, 154.1, 174.9 ppm; Anal. Calcd. for C_16_H_20_ClNS (293.10): C, 65.40; H, 6.86; N, 4.77; Found: C, 65.44; H, 6.86; and, N, 4.76.

#### 3.1.6. General Method for the Synthesis of 5-Trifluoromethylbenzothiazoles **16**

2-Amino-4-trifluoromethylthiophenol hydrochloride (5 mmol, 1.148 g) was dissolved in 30 mL of distilled water and an equimolar amount of DBU (5 mmol, 0.745 mL) was added. The mixture was stirred with magnetic stirrer for 1 h and was then extracted with chloroform (3 × 10 mL). The combined organic layers were dried over anhydrous MgSO_4_. The drying agent was filtered off and the solvent evaporated. To the free thiophenol, the appropriate carboxylic acid (5 mmol) was added and the reagents were heated with silica gel (1.5 g) and methanesulfonic acid (5 mL) at 140 °C for 72 h, then treated as in procedure described for benzothiazoles **14**, **15,** and purified by column chromatography with chloroform as the eluent.

*2-(3-Cyclohexylpropyl)-5-(trifluoromethyl) benzo[d]thiazole* (**16**). Starting from 4-cyclohexylbutyric acid (0.850 g), the title compound **16** was obtained as pink crystals (0.551 g, 34%): m.p. 33–35 °C; IR (KBr): 3056 (ν C–H), 2926, 2846 (ν C–H), 1509, 1422 (ν C=C), 1329 (ν F_3_C–Ar), 1173, 1149, 1123 (ν C–F), 918, 813 (γ C–H) cm^−1^; ^1^H-NMR (200 MHz, DMSO-*d*_6_): δ 0.79–1.27 (m, 8H, 4CH_2_), 1.42–1.88 (m, 7H, 6H 3CH_2_ and 1H CH), 3.11 (t, 2H, CH_2_, *J* = 7 Hz), 7.71 (d, 1H, ArH, *J* = 9 Hz), 8.27–8.32 (m, 2H, ArH) ppm; ^13^C-NMR (50 MHz, DMSO-*d*_6_): δ 26.1 (2C), 26.5, 26.6, 27.6, 33.1 (2C), 34.1, 36.5, 37.1, 119.2, 121.1, 123.9, 127.0, 139.2, 152.7, 175.3 ppm; Anal. Calcd. for C_17_H_20_F_3_NS (327.13): C, 62.36; H, 6.16; N, 4.28; Found: C, 62.48; H, 6.13; and, N, 4.16.

#### 3.1.7. General Method for the Synthesis of 2-Pyridinebenzimidazoles **17**–**20**

4-Substituted picolinothioamide (1 mmol) and the 4,5-dimethyldiamine (1.3 mmol) were refluxed in ethylene glycol (3 mL) until hydrogen sulfide evolution ceased (3–5 h). Then, ice (20 g) was added and the precipitated product was collected by filtration and purified by recrystallization with the addition of activated carbon. 

*5,6-Dimethyl-2-(pyridin-2-yl)-1H-benzo[d]imidazole* (**17**) Starting from pyridine-2-carbothioamide (0.138 g), the title compound **17** was obtained as light brown crystals (0.156 g, 70%): m.p. 198–200 °C; IR (KBr): 3373 (ν N–H), 2921 (ν C–H), 1593 (δ N–H), 1451 (ν C=C), 995 (γ C–H) cm^−1^; ^1^H-NMR (200 MHz, DMSO-*d*_6_): δ 2.37 (s, 6H, CH_3_), 7.34–7.50 (m, 3H, 2ArH and 1pyridine), 7.97 (d, 1H, pyridine, *J* = 7 Hz), 8.29 (d, 1H, pyridine, *J* = 8 Hz), 8.70 (d, 1H, pyridine, *J* = 9 Hz), 12.87 (s, 1H, NH) ppm; ^13^C-NMR (50 MHz, DMSO-*d*_6_): δ 20.33 (2C), 112.00, 118,5, 121.40 (2C), 124.57 (2C), 137.65 (2C), 149.08, 149.48 (2C), 150.15 ppm; Anal. Calcd. for C_14_H_13_N_3_ (223.11): C, 75.31; H, 5.87; N, 18.82; Found: C, 75.28; H, 5.73; and, N, 18.43.

*5,6-Dimethyl-2-(4-(pyrrolidin-1-yl)pyridin-2-yl)-1H-benzo[d]imidazole* (**18**) Starting from 4-(pyrrolidin-1-yl)pyridine-2-carbothioamide (0.207 g), the title compound **18** was obtained as white crystals (0.250 g, 86%): m.p. 293–294 °C; IR (KBr): 3050 (ν C_Ar_–H), 2970, 2849 (ν C–H), 1606 (δ N–H), 1513, 1458 (ν C=C), 1012, 985, 853 (γ C–H) cm^−1^; ^1^H-NMR (500 MHz, DMSO-*d*_6_): δ 1.98 (t, 4H, CH_2_, *J* = 6 Hz), 2.29 (s, 6H, CH_3_), 3.33–3.36 (m, 4H, CH_2_), 6.53 (dd, 1H, pyridine, *J*_1_ = 2 Hz, *J*_2_ = 3 Hz), 7.25 (s, 1H, ArH), 7.35 (d, 1H, pyridine, *J* = 2 Hz), 7.41 (s, 1H, ArH), 8.19 (d, 1H, pyridine, *J* = 6 Hz), 12.62 (s, 1H, NH) ppm; Anal. Calcd. for C_18_H_20_N_4_ (292.19): C, 73.94; H, 6.89; N, 19.16; Found: C, 73.76; H, 6.87; and, N, 18.99.

*4-(2-(5,6-Dimethyl-1H-benzo[d]imidazol-2-yl)pyridin-4-yl)morpholine* (**19**) Starting from 4-morpholinopyridine-2-carbothioamide (0.223 g), the title compound **19** was obtained as white solid (0.290 g, 94%): m.p. >300 °C; IR (KBr): 2963, 2854 (ν C–H), 1598 (δ N–H), 1489, 1445 (ν C=C), 1118 (δ C–H), 989, 946 (γ C–H) cm^−1^; ^1^H-NMR (500 MHz, DMSO-*d*_6_): δ 2.30 (s, 6H, CH_3_), 3.36–3.38 (m, 4H, CH_2_), 3.74 (t, 4H, CH_2_, *J* = 4 Hz), 6.92 (dd, 1H, pyridine, *J*_1_ = 2 Hz, *J*_2_ = 3 Hz), 7.26 (s, 1H, ArH), 7.41 (s, 1H, ArH), 7.70 (d, 1H, pyridine, *J* = 2 Hz), 8.30 (d, 1H, pyridine, *J* = 6 Hz), 12.69 (s, 1H, NH) ppm; Anal. Calcd. for C_18_H_20_N_4_O (308.16): C, 70.11; H, 6.54; 18.17; Found: C, 70.47; H, 6.32; and, N, 18.15.

*5,6-Dimethyl-2-(4-(4-phenylpiperazin-1-yl)pyridin-2-yl)-1H-benzo[d]imidazole* (**20**) Starting from 4-(4-phenylpiperazin-1-yl)pyridine-2-carbothioamide (0.298 g), the title compound **20** was obtained as light brown solid (0.350 g, 91%): m.p. 267–268 °C; IR (KBr): 3413 (ν N–H), 1598 (δ N–H), 1494, 1447 (ν C=C), 1228 (δ C–H) cm^−1^; ^1^H-NMR (200 MHz, DMSO-*d*_6_): δ 2.31–2.34 (m, 6H, CH_3_), 3.39 (t, 8H, CH_2_, *J* = 21 Hz), 6.81–7.46 (m, 8H, 6ArH and 2pyridine), 7.77 (s, 1H, ArH), 8.30 (d, 1H, pyridine, *J* = 5 Hz); 12.75 (s, 1H, NH) ppm; Anal. Calcd. for C_24_H_25_N_5_ (383.21): C, 75.17; H, 6.57; N, 18.26; Found: C, 75.19; H, 6.82; and, N, 17.87.

### 3.2. Antimycobacterial Activity Assay

The synthesized compounds were examined in vitro for their tuberculostatic activity against the *Mycobacterium tuberculosis* H_37_Rv and “wild” strain that was isolated from tuberculosis patients (Spec. 210) resistant to PAS, INH, ETB and RMP. M. tuberculosis strain H37RV ATCC 25618 was purchased from LGC Standards (Middlesex, UK), which is an intermediary for the sale of reference strains from the American Type Culture Colection (ATCC). Tuberculosis bacteria cultures and sputum samples from tuberculosis positive patients, were cultured, stored, prepared, and used for experiments in microbiological laboratory of National Institute of Tuberculosis and Lung Diseases in Warsaw, Poland. Investigations were performed by a classical test-tube method of successive dilution in Youmans’ modification of the Proskauer and Beck liquid medium containing 10% of bovine serum [35,36]. Bacterial suspensions were prepared from 14 days old cultures of slowly growing strains and from 48 h old cultures of saprophytic strains [37,38]. Solutions of compounds in ethylene glycol were tested. Stock solutions contained 10 mg of compounds in 1 milliliter. Dilutions (in geometric progression) were prepared in Youmans’ medium. The medium containing no investigated substances and containing INH, PZA or RMP as reference drugs were used for comparison. Incubation was performed at a temperature of 37 °C. The MIC values were determined as minimum concentration inhibiting the growth of tested tuberculosis strains in relation to the probe with no tested compound. The influence of the compound on the growth of bacteria at a certain concentration 3.1, 6.2, 12.5, 25, 50, and 100 µg/mL were evaluated.

### 3.3. Quantum Chemical Calculations

Quantum chemical calculations were carried out to study the molecular geometry and electronic structure of benzimidazoles **GK-151B**, **4**, **6**, and benzoxazole **11** using the Gaussian 03W software (Gaussian 03, Revision A.1., Gaussian, Inc., Wallingford, CT USA) The full optimized geometries of both compounds in vacuum were calculated by density functional theory-B3LYP method using diffuse functions 6-31G(d) basis set. Three-dimensional structure of benzimidazoles **1**–**3**, **MC-9,** and **GK-151B** was determined by ab initio calculation using Gamess-US [39] software (Iowa State University, Ames, IA, USA). DFT/M06-2X functional with 6-311G(d,p) basis set was used.

## 4. Conclusions

In conclusion, a series of 20 derivatives of benz[*d*]imidazole, benzo[*d*]oxazole, and benzo[*d*]thiazole were successfully synthesized. Their tuberculostatic activity in vitro was evaluated against *M. tuberculosis* H_37_Rv and Spec. 210 strains. The activity of the obtained benzimidazoles toward *M. tuberculosis* was good to weak (3.1–100 µg/mL). Whereas, the benzoxazoles and benzothiazoles exhibited much lower activity than that was observed previously for their benzimidazole analogs. The determined MIC values for tested compounds were in the range of 25–100 µg/mL, while for the most active benzimidazole analogs, it was at the level of 0.75 µg/mL [16]. The quantum chemical calculations performed for selected benzazoles demonstrated significant differences in the distribution of electrostatic potential. These results prove that benzimidazole system is essential for the antituberculosis activity that was associated with the presence of the imine nitrogen atom in N-1 position. Its replacement by an oxygen or sulfur atom results in a dramatic decrease of activity, which probably is a consequence of the weaker interaction with the cell target. In addition, the parity of the alkyl substituent at the C-2 position influences the spatial arrangement of the molecule. Compounds without linker or with even alkyl chain exhibited higher antitubercular activity, which may be associated with better alignment to the cell target. The cyclohexyl or pyridine ring promotes the occurrence of tuberculostatic activity, while no activity was observed in the presence of phenyl ring.

## Figures and Tables

**Table 1:** In vitro tuberculostatic activity of compounds **1**–**20**.

Compd.	MIC ^a^ [µg/mL (µM)]		Compd.	MIC ^a^ [µg/mL (µM)]	
	*M. tuberculosis* ^c^			*M. tuberculosis* ^c^	
	H_37_Rv	Spec. 210		H_37_Rv	Spec. 210
**1**	3.1 (13)	**3.1** (**13**)	**13**	100 (361)	100 (361)
**2**	**6.25** (**25**)	**6.25** (**25**)	**14**	100 (358)	100 (358)
**3**	**1.5** (**5**)	**1.5** (**5**)	**15**	100 (341)	100 (341)
**4**	**3.1** (**11**)	**3.1** (**11**)	**16**	100 (306)	100 (306)
**5**	25 (96)	12.5 (48)	**17**	12.5 (56)	12.5 (56)
**6**	50 (190)	25 (95)	**18**	12.5 (43)	25 (86)
**7**	25 (89)	12.5 (44)	**19**	25 (81)	25 (81)
**8**	>100 (>409)	>100 (>409)	**20**	12.5 (33)	12.5 (33)
**9**	50 (225)	50 (225)	**INH** ^b^	0.125 (0.91)	12.5 (91)
**10**	25 (91)	25 (91)	**PZA**	25 (203)	>400 (3249)
**11**	50 (194)	50 (194)	**RMP**	1.2 (1.4)	2.5 (2.8)
**12**	50 (190)	50 (190)			

The values obtained for the most potent compounds are marked in bold. ^a^ Minimum inhibitory concentrations for mycobacterial strains was determined by two-fold classical test-tube method of successive dilution. ^b^ INH isoniazid; PZA pyrazinamide; RMP rifampicin; ^c^ *M. tuberculosis* H_37_Rv, Spec. 210.

**Table 2:** Calculated values of absolute energy (E), dipole moment (μ) and partition coefficient (log P) of benzimidazoles **GK-151B**, **4**, **6**, and benzoxazole **11**.

Compd.	MIC (µg/mL)	µ (Debye)	log P
**GK-151B**	0.75	3.28	4.95
**4**	3.1	3.57	5.95
**6**	25-50	4.92	5.13
**11**	50	0.93	5.02

## Supplementary Materials

Supplementary Materials are available online.
