# Supplementary material for: Synthesis and Tuberculostatic Activity Evaluation of Novel Benzazoles with Alkyl, Cycloalkyl or Pyridine Moiety
Source: Molecules. 2018 Apr 23;23(4):985. doi: 10.3390/molecules23040985 (PMC6017942; doi:10.3390/molecules23040985)
Supplement: Supplementary file 1 [file molecules-23-00985-s001.pdf]

Supplementary materials

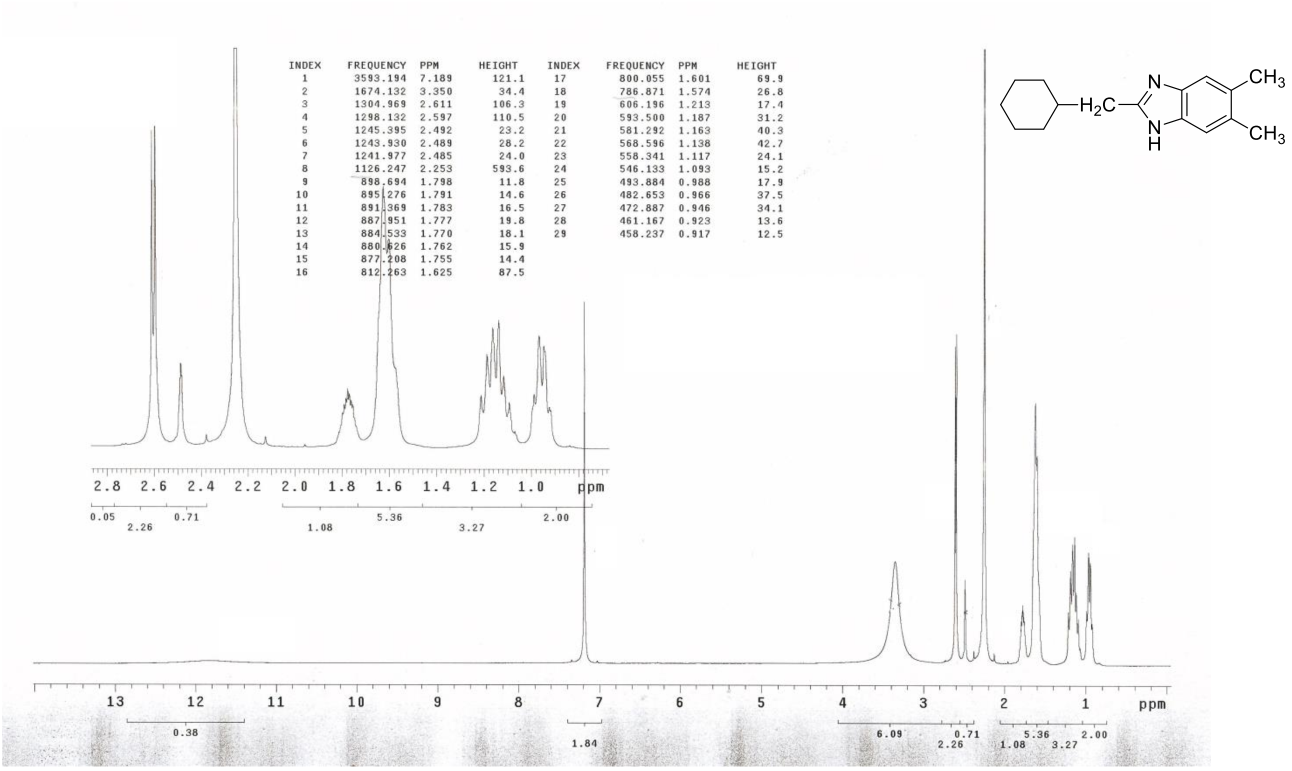

Figure S1. ^{1}H-NMR of compound **2** (500 MHz, DMSO-*d6*)

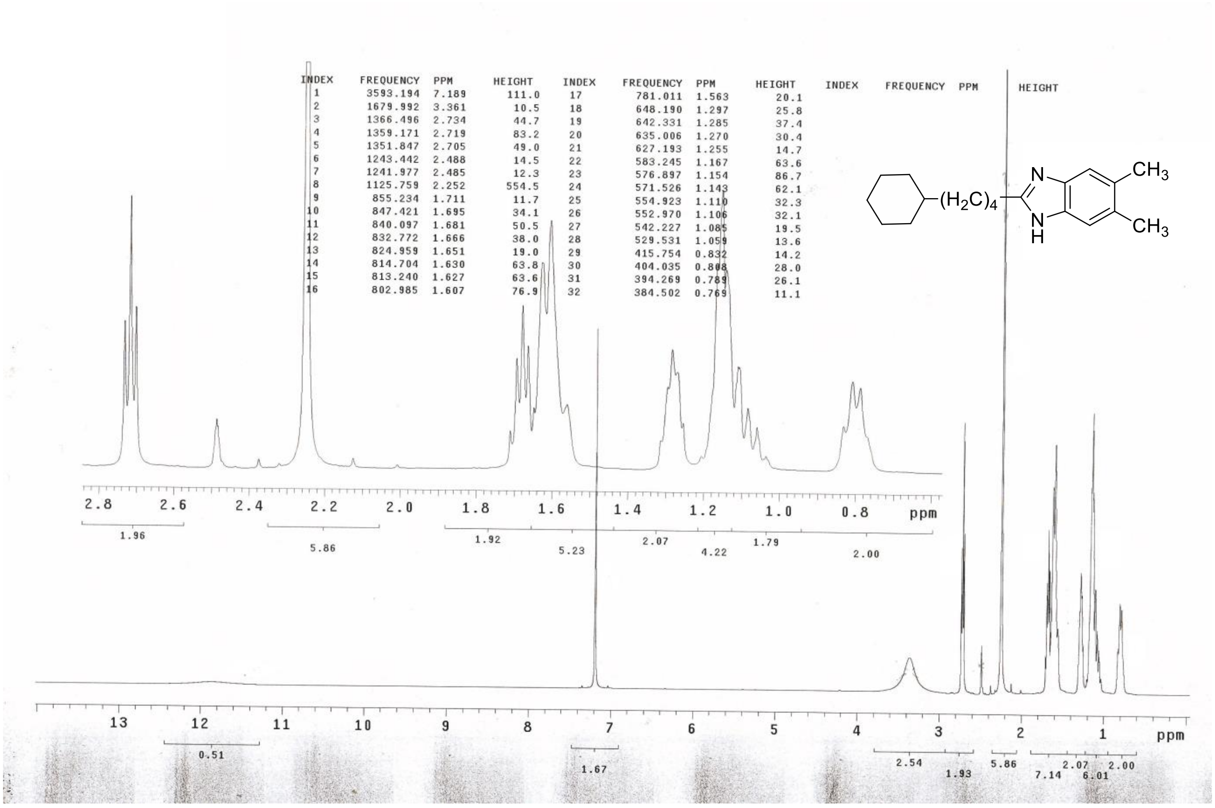

Figure S2. ^{1}H-NMR of compound **3** (500 MHz, DMSO-*d6*)

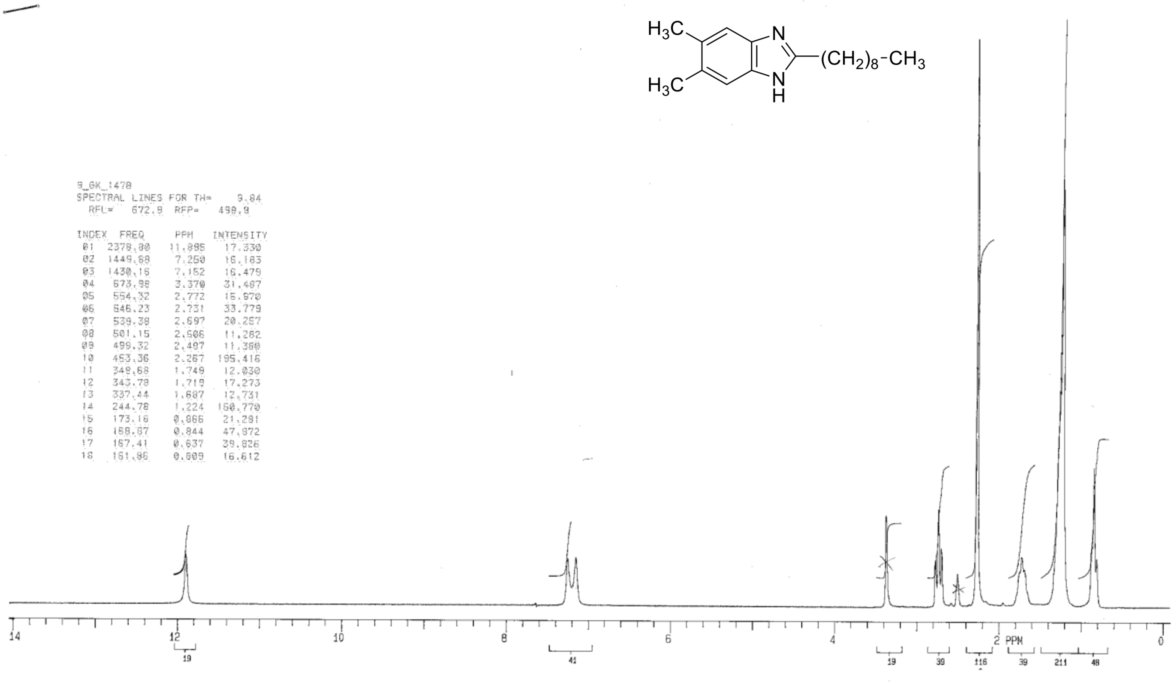

Figure S3 ^{1}H-NMR of compound **4** (200 MHz, DMSO-*d6*)

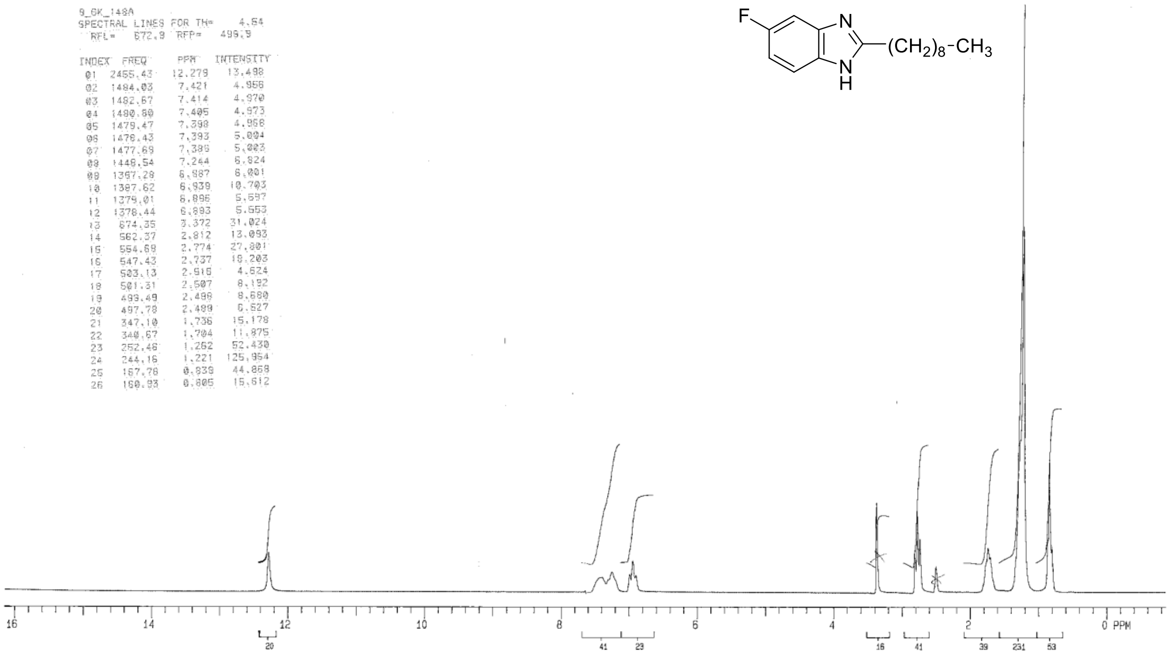

Figure S4 ^{1}H-NMR of compound **6** (200 MHz, DMSO-*d6*)

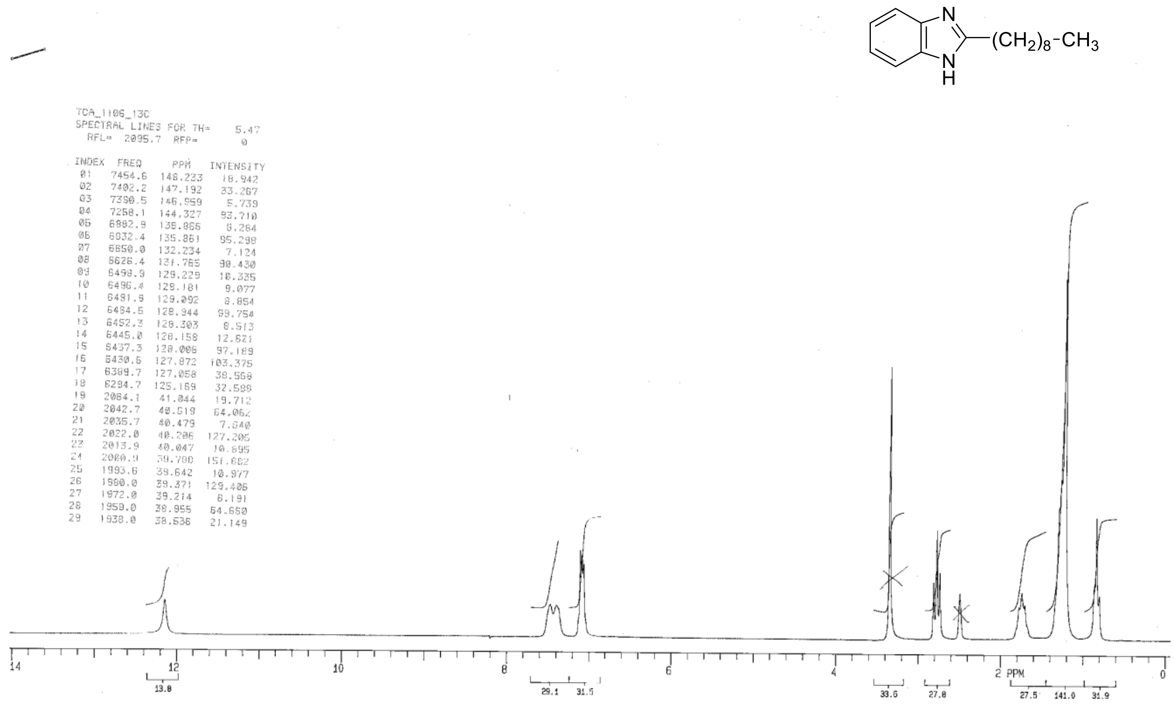

Figure S5 ^{1}H-NMR of compound **8** (200 MHz, DMSO-*d6*)

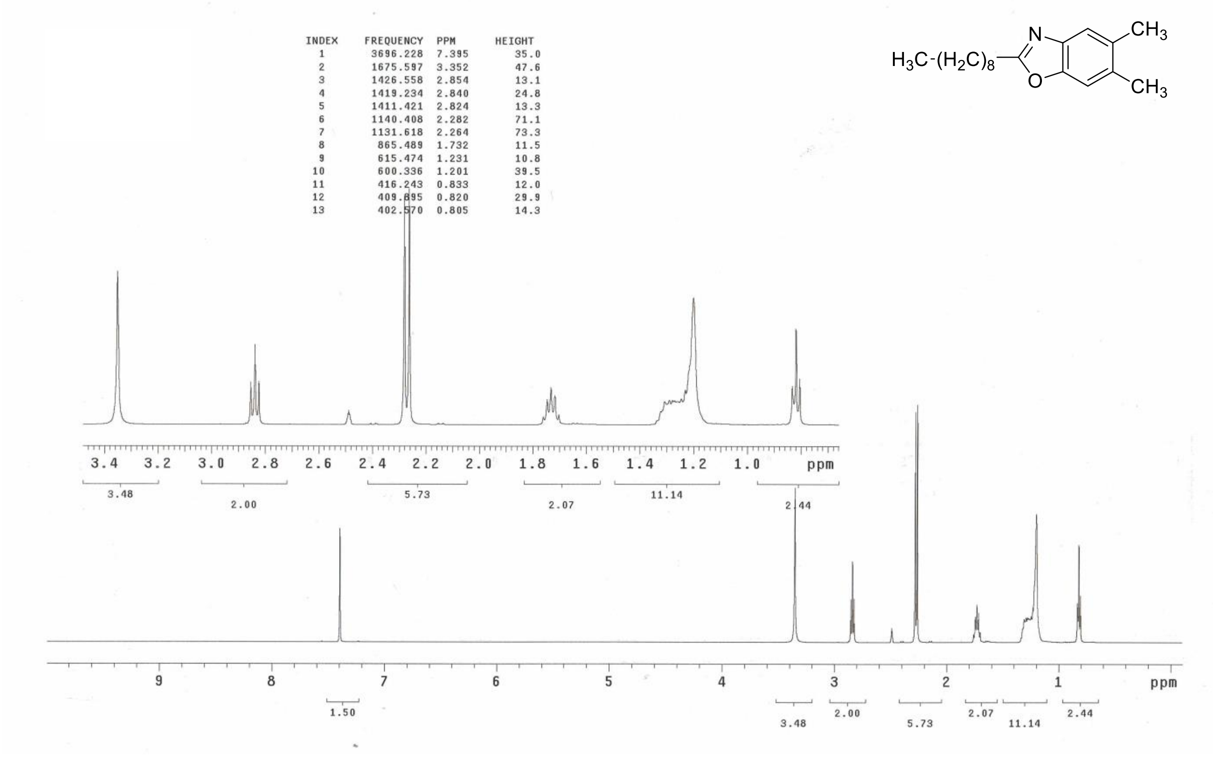

Figure S6. ^{1}H-NMR of compound **10** (500 MHz, DMSO-*d6*)

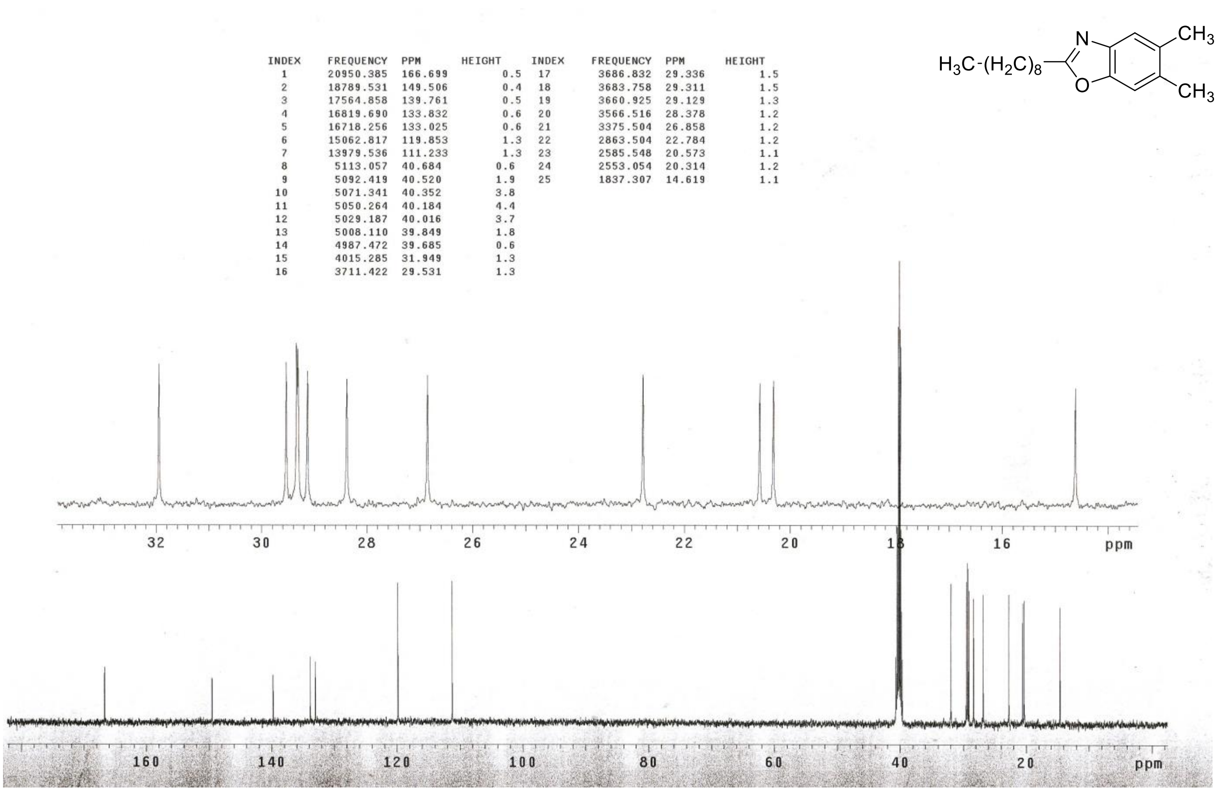

Figure S7. ^{13}C-NMR of compound **10** (500 MHz, DMSO-*d6*)

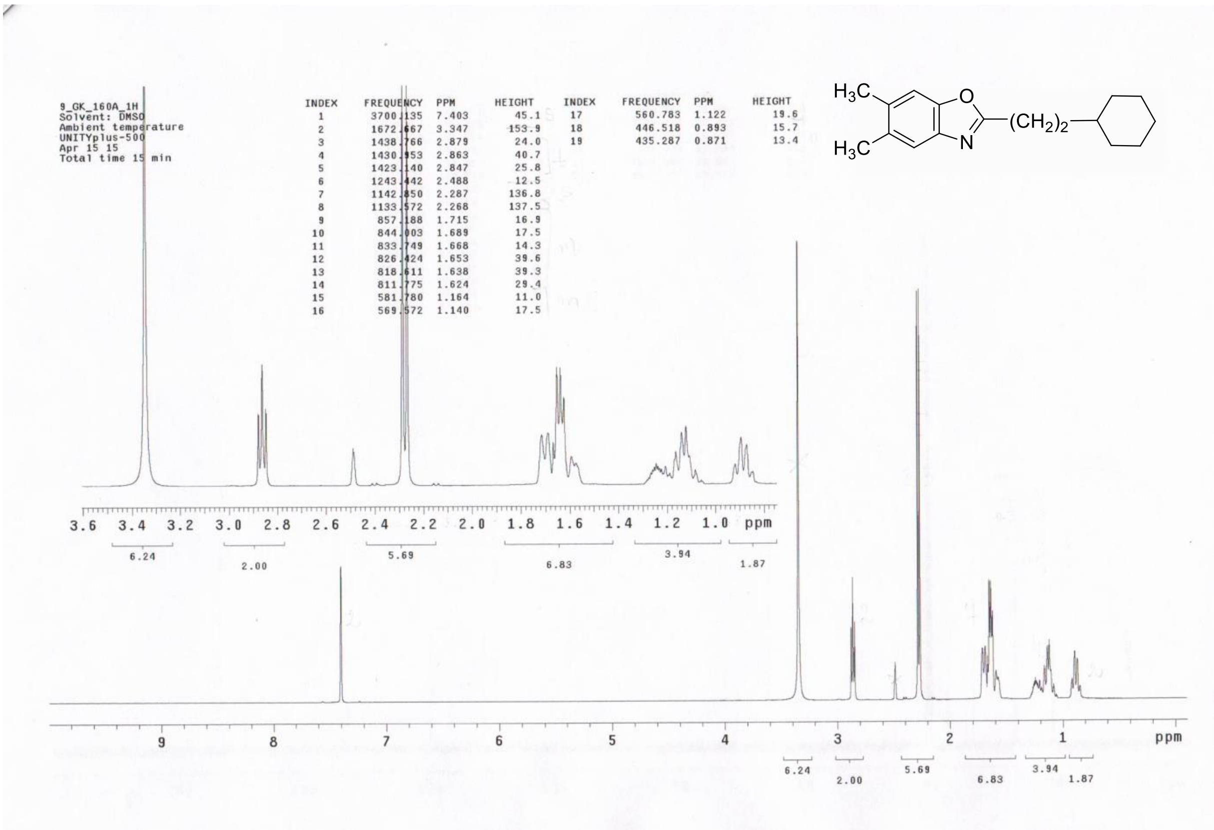

Figure S8. ^{1}H-NMR of compound **11** (500 MHz, DMSO-*d6*)

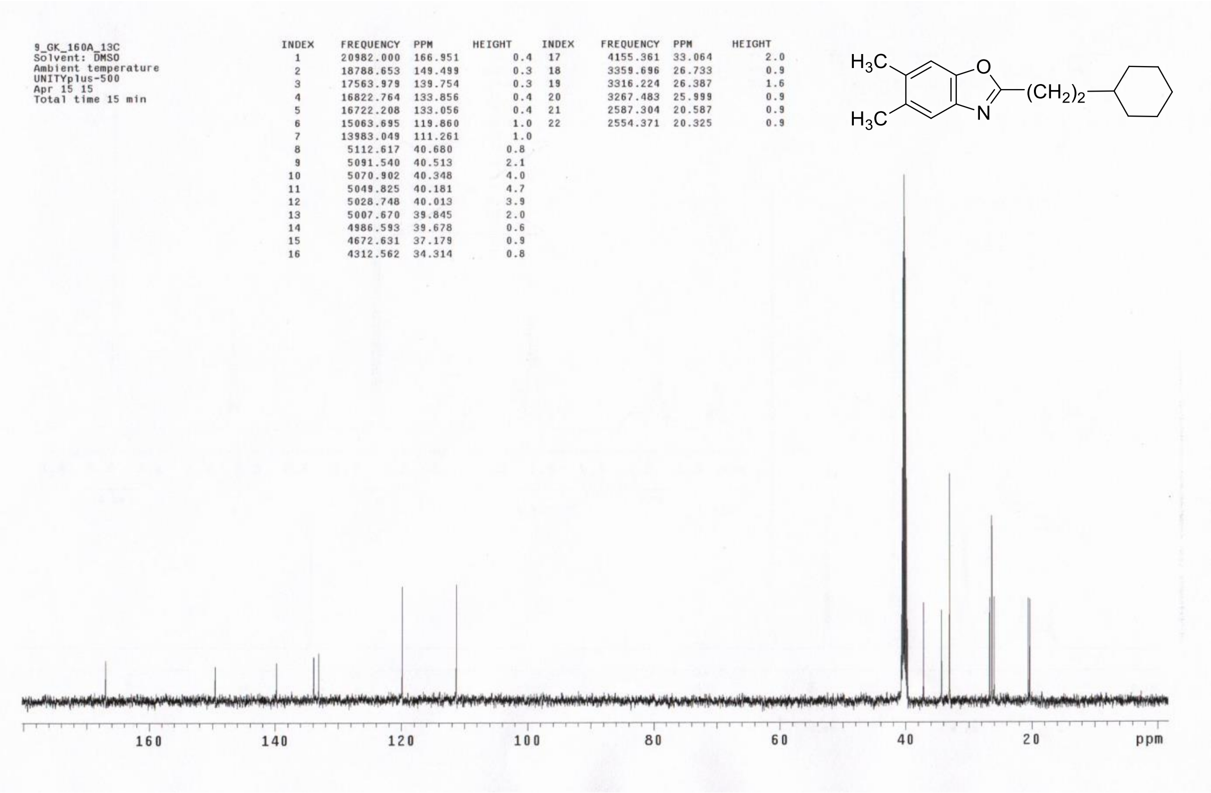

Figure S9. ^{13}C-NMR of compound **11** (500 MHz, DMSO-*d6*)

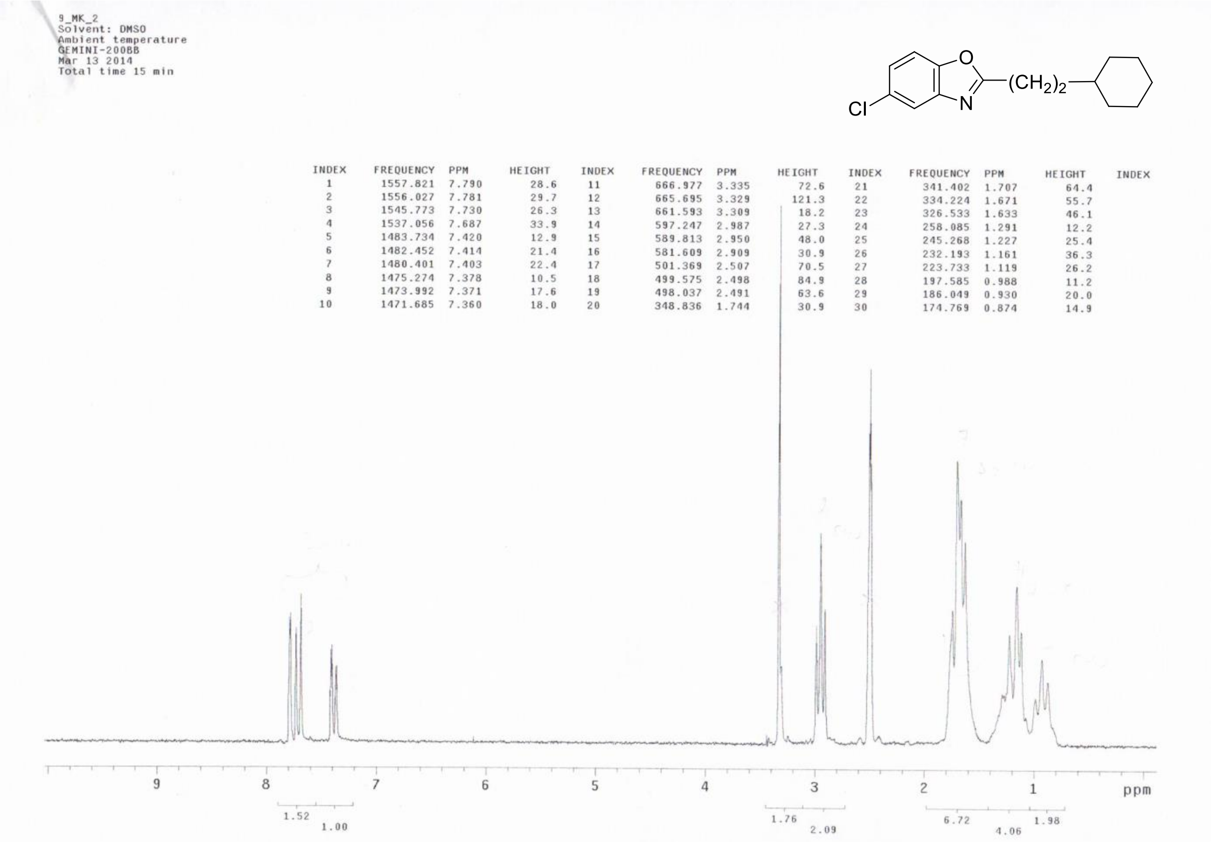

Figure S10. ^{1}H-NMR of compound **12** (500 MHz, DMSO-*d6*)

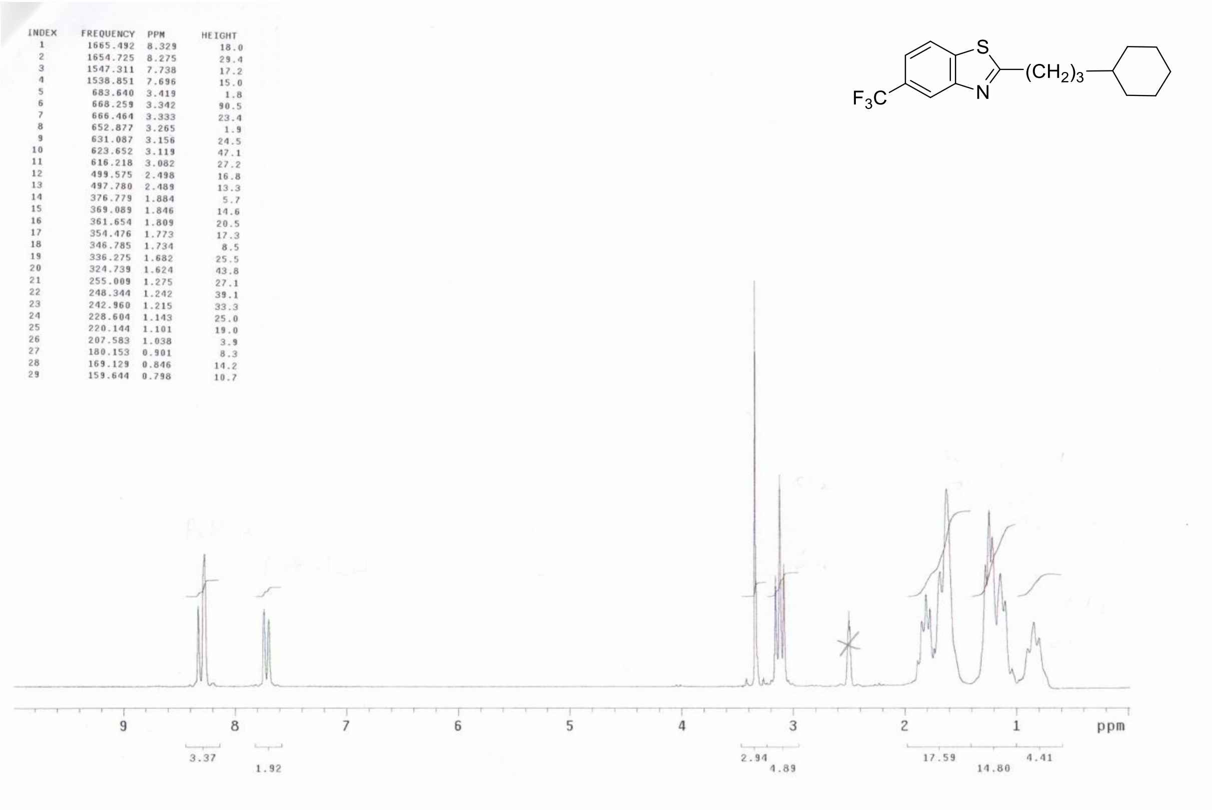

Figure S11. ^{1}H-NMR of compound **16** (500 MHz, DMSO-*d6*)

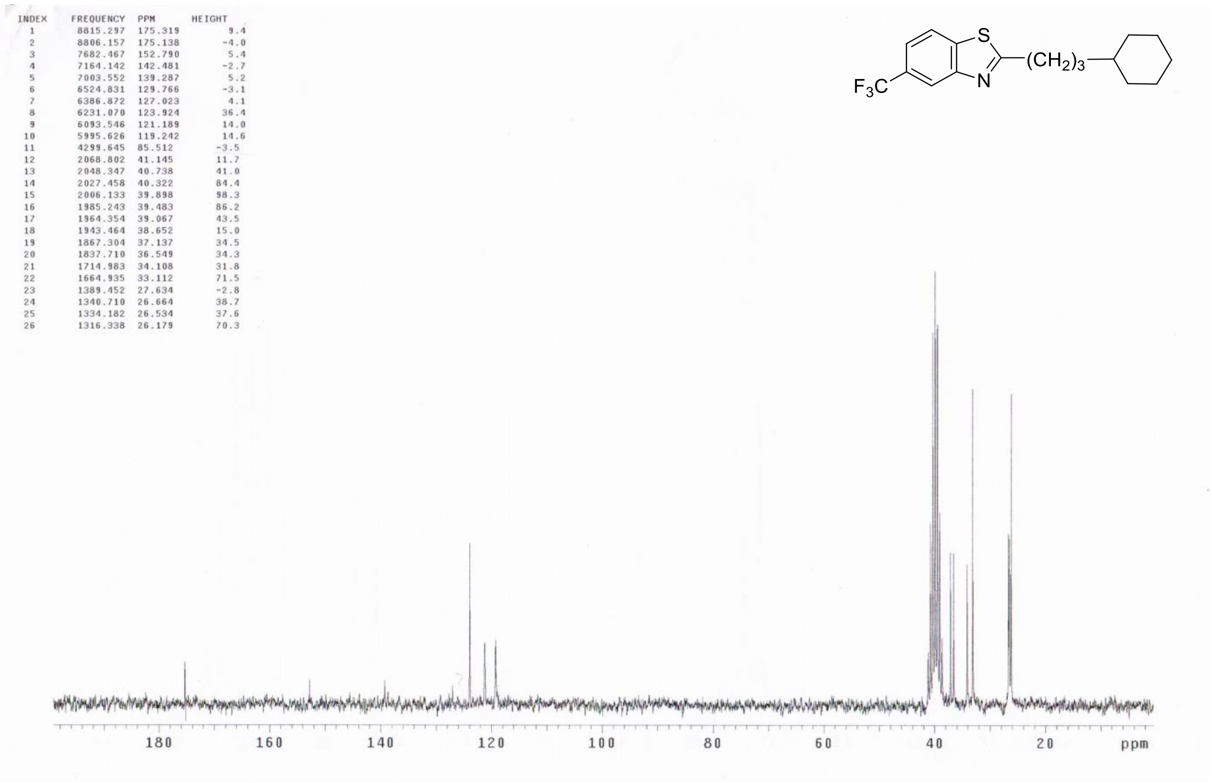

Figure S12. ^{13}C-NMR of compound **16** (500 MHz, DMSO-*d6*)

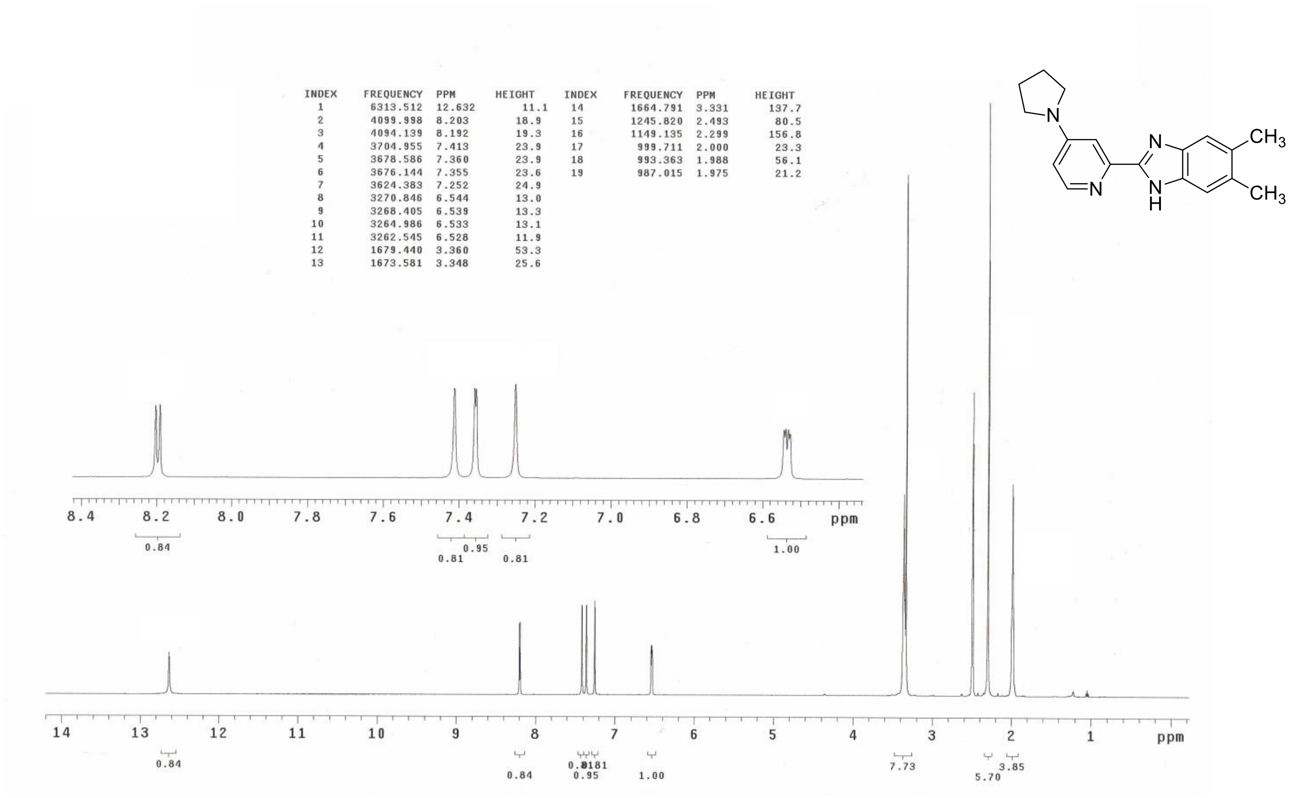

Figure S13. ^{1}H-NMR of compound **18** (500 MHz, DMSO-*d6*)

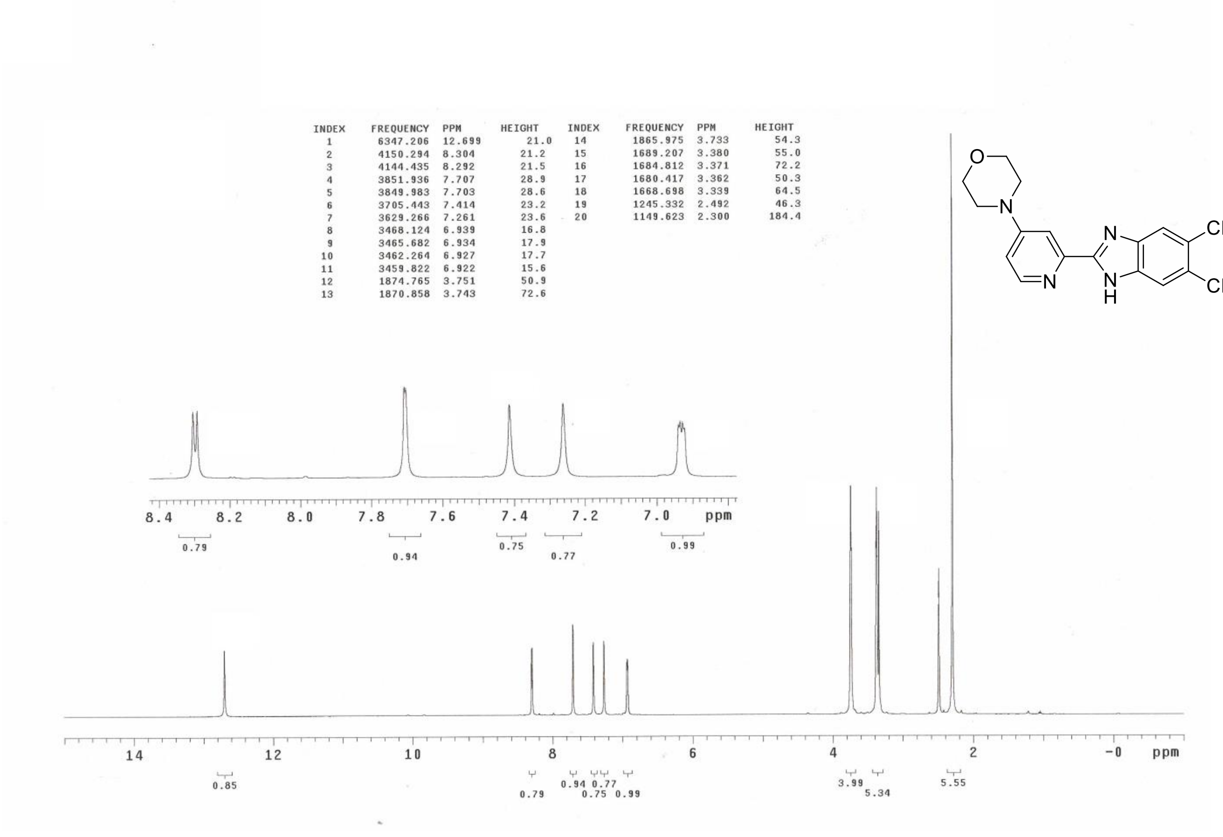

Figure S14. ^{1}H-NMR of compound **19** (500 MHz, DMSO-*d6*)
